# Nonatonic Obstetric Haemorrhage: Effectiveness of the Nonpneumatic Antishock Garment in Egypt

**DOI:** 10.5402/2011/179349

**Published:** 2011-08-10

**Authors:** Mohamed M. F. Fathalla, Mohammed Mourad Youssif, Carinne Meyer, Carol Camlin, Janet Turan, Jessica Morris, Elizabeth Butrick, Suellen Miller

**Affiliations:** ^1^Department of Obstetrics and Gynaecology, Faculty of Medicine, Assiut University Women's Health Center, P.O. Box 30, Assiut, Egypt; ^2^Department of Obstetrics and Gynaecology, El Galaa Maternity Teaching Hospital, Cairo, Egypt; ^3^Department of Obstetrics, Gynaecology and Reproductive Sciences, University of California, San Francisco, CA, USA

## Abstract

The study aims to determine if the nonpneumatic antishock garment (NASG), a first aid compression device, decreases severe adverse outcomes from nonatonic obstetric haemorrhage. Women with nonatonic aetiologies (434), blood loss > 1000 mL, and signs of shock were eligible. Women received standard care during the preintervention phase (226) and standard care plus application of the garment in the NASG phase (208). Blood loss and extreme adverse outcomes (EAO-mortality and severe morbidity) were measured. Women who used the NASG had more estimated blood loss on admission. Mean measured blood loss was 370 mL in the preintervention phase and 258 mL in the NASG phase (*P* < 0.0001). EAO decreased with use of the garment (2.9% versus 4.4%, (OR 0.65, 95% CI 0.24–1.76)). In conclusion, using the NASG improved maternal outcomes despite the worse condition on study entry. These findings should be tested in larger studies.

## 1. Introduction

Obstetric haemorrhage is the most common cause of maternal mortality in the developing world [1]. In Egypt, postpartum haemorrhage is the most frequent cause of maternal mortality, constituting 33.4% of maternal mortality [2]. Advances in the prevention and management of atonic postpartum haemorrhage include new uterotonics such as misoprostol, new administration technologies for established uterotonics, such as oxytocin in Uniject, balloon compression devices, and uterine compression sutures [3]. However, these advances do not benefit women with nonatonic aetiologies. Previous estimates considered uterine atony to be responsible for the majority of postpartum haemorrhage mortality [4]. In other studies of severe haemorrhage, nonatonic aetiologies were found to be more common than expected [5]. 

The nonpneumatic antishock garment (NASG) (Zoex Corporation, Ashland, Ore, USA) is a neoprene and Velcro lower-body first aid pressure device for hypovolaemic shock made up of nine horizontal segments: three per leg (ankle, calf, and thigh), one for the pelvis, and a larger segment with a small foam compression ball for the abdomen. This device reverses shock by decreasing the transmural pressure and radius of the blood vessels in the lower body, thus decreasing blood flow to the abdomen and lower body and redirecting blood to the core organs. When a woman is experiencing obstetric haemorrhage, this device can restore her consciousness, pulse, and blood pressure, and it can buy her time to receive definitive therapy [6]. The NASG is relatively low cost at $170 USD per garment which can be used approximately 40 times.

The NASG is uniquely applicable as a first aid device in settings where delays in management are common. It also allows complete access to the perineal area enabling vaginal procedures without unfastening the garment and has an easily opened abdominal segment enabling surgery while the rest of the garment remains closed. 

We have previously published the outcomes of a pilot study in Egypt (*n* = 364) [5], an interim analyses of a small (*n* = 169) study at one facility in Nigeria [7] and a larger study in Egypt (*n* = 990) [8]. This is a subgroup analysis of the latter Egypt study which examines women with obstetric haemorrhage and shock due to nonatonic aetiologies treated with standard protocol versus nonatonic aetiologies treated with standard protocol plus NASG.

## 2. Methods

In this study, 434 women were recruited from the emergency admissions of two referral hospitals in Egypt; Assiut University Women's Health Centre (13500 deliveries annually) and El Galaa Teaching Hospital in Cairo (20000 deliveries annually). Both facilities receive referrals from home deliveries and lower level facilities, and both are staffed by consulting obstetricians and residents.

### 2.1. Settings and Participants

This study was conducted from June 2006 to May 2008. Women were eligible regardless if they began haemorrhaging outside the facility and were transferred in or began haemorrhaging in the facility. Included in this analysis were women with obstetric haemorrhage and shock due to any of the following aetiologies: ectopic gestation, trophoblastic disease of pregnancy, placenta praevia, accreta or abruption, ruptured uterus, or vaginal or cervical lacerations. All women had an estimated blood loss > 1000 mL, a pulse >100 beats per minute, or systolic blood pressure (SBP) < 100 mmHg on study admission. Clinician data collectors were trained on estimation of concealed blood using the patient's vital signs and symptoms as indicators [9–11]. Estimation of revealed blood loss was done using visual estimation and questioning of the patient. The following conditions were contraindications to NASG use and study exclusion: heart disease, pulmonary hypertension, a viable foetus in utero (e.g., some women with placenta previa), and the presence of bleeding above the diaphragm. 

The 434 women who had nonatonic haemorrhage aetiologies represent 44% of the total women included in the larger study. Of those, 226 women were in the preintervention phase and 208 in the NASG phase. In the preintervention phase, women were treated following standardized evidence-based protocols that were prepared and approved by the Egyptian Ministry of Health and Population [12]. In the NASG phase, 208 women were treated with the standardized protocol plus application of the NASG.

Data was collected by staff in the facilities who were trained on the standardized protocol, blood collection and measurement, NASG use, and completion of data collection forms. The volume of external blood loss was calculated using a plastic blood collection drape (BRASSS-V Fixable Drape Madurai, India) that is snugly tied around the patient's waist and the collection bag is opened underneath the woman. Blood loss suctioned or mopped with gauze or towels during surgery was also measured and recorded.

### 2.2. Informed Consent

The study protocol was approved by the University of California, San Francisco (UCSF) Committee on Human Research (CHR) (approval number H6899-23524) and given ethical clearance by the Institutional Review Boards of Assiut University Faculty of Medicine and El Galaa Teaching Hospital, Cairo. In the preintervention phase women gave informed consent to use their data. Women in the intervention phase provided written informed consent for the NASG and the use of their data. A US Federal waiver of consent/authorization for minimal risk research was obtained (45 CFR 46, 45 CFR 164.512) so that women who were unconscious or confused at study entry were enrolled and treatment was begun, but their data would only be used if they gave informed consent after they regained normal sensorium or if a relative gave consent on their behalf.

### 2.3. Clinical Protocols

Following enrolment in the study, women's vital signs and blood loss were monitored while the aetiology of the haemorrhage was identified and resuscitation and treatment initiated. The women remained in the study until vital signs had stabilized (SBP > 100, pulse < 100) for a minimum of two hours and blood loss had decreased to approximately 25–50 mL per hour. Standard haemorrhage/shock protocols at these facilities included: administration of crystalloid intravenous fluids (≥1500 mL in the first hour), vaginal procedures, and provision of blood transfusions and/or surgery as necessary. The NASG was left completely in place during vaginal procedures. If laparotomy was required, abdominal and pelvic segments were opened immediately prior to making the incision and then replaced as soon as the surgery was completed.

### 2.4. Outcomes

The primary outcome was extreme adverse outcomes which include mortality and severe morbidity. Morbidities were defined as organ system dysfunctions related to severe obstetric hemorrhage and included: acute respiratory distress syndrome (impairment of respiratory function needing ventilation, oxygen supplementation, or decreased physical activity level as compared to pre-pregnancy), cerebral impairment (seizures, unconsciousness, or cognitive/motor loss), renal failure (creatinine > 1.5 mg/dL or increased >1.0 mg/dL above baseline, oliguria; <120 mL output in 4-hour intervals), and heart failure (impairment of cardiac function according to New York Heart Disease Classification) [13]. The secondary outcomes were cumulative blood loss measured hourly after study admission with the calibrated drape and morbidity and mortality as individual variables.

### 2.5. Severity of Condition

The indicator of the severity of the woman's condition at study entry was mean arterial pressure (MAP = [2*diastolic blood pressure + systolic blood pressure]/3) on study admission. Women with MAP < 60 mmHg were considered to be in more severe shock.

### 2.6. Data Collection

Hospital residents and nurses were trained in the standardized protocol for management of obstetric haemorrhage and shock, blood collection and measurement, and completion of data collection forms. Prior to the intervention phase, the staff was trained to use the NASG. All data were collected by the clinicians as they cared for or immediately after caring for the patient in shock. All data collection forms were reviewed by Egyptian Principal Investigators and quality control was conducted regularly during each study phase to resolve any disagreement or inconsistency. Forms were sent electronically via a datafax system (Clinical DataFax Systems Inc., ON, Canada) to statisticians at the University of California, San Francisco (UCSF) to be entered and analysed.

### 2.7. Statistical Analysis

Participants' demographic characteristics, condition on study entry, and treatment received in the two study phases were compared using *t*-tests (assuming unequal variances in the two phase populations) and chi-square tests of independence. Fisher's exact tests were used for small cell sizes. Relative risks (RR) and 95% confidence intervals were computed for the primary outcome, EAOs, and for the secondary outcomes, mortality and severe morbidity. Mean measured volume of blood loss in the drape was compared across phases with *t*-tests.

## 3. Results

### 3.1. Baseline Characteristics

Women in the two study phases were comparable regarding demographic characteristics and duration of gestation. In the preintervention phase, there were more women with placenta praevia (*P* = 0.014). In the NASG phase, there were more women with vaginal or cervical lacerations (*P* = 0.001) (see Table 1).

### 3.2. Condition on Study Entry

Women in the NASG phase had significantly more estimated revealed and concealed blood loss (Table 1). In addition, more women in the NASG phase were in severe condition (MAP < 60 mmHg) upon study entry: 24.5% as compared to 15.6% in the preintervention phase.

### 3.3. Treatment

Treatment variables (Table 2) show significantly fewer women in the NASG phase receiving either ≥1500 mL crystalloid fluids or a blood transfusion in the first hour (*P* < 0.001). By the end of the study admission, there was no difference in the proportion of women who received a blood transfusion.

### 3.4. Outcomes

Mean measured blood loss was significantly lower in the NASG phase (*P* < 0.001). There were more EAOs in the preintervention phase although the difference was not statistically significant. More women died in the NASG phase while fewer women experienced severe morbidities; neither were statistically significant (Table 3). Use of the NASG did not confer any significant increase in the side effects that were investigated, in fact there was significantly less nausea and vomiting in the NASG phase (Table 4). 

## 4. Discussion

### 4.1. Main Findings

The study results agree with previous research that the NASG is useful for patients with obstetric haemorrhage of nonatonic aetiologies. This study found the device to be safe with no side effects. The effect of NASG on the combined variable, EAO, and on severe morbidity for women with nonatonic aetiologies confirms previous reports of the benefit of NASG for all obstetric haemorrhage aetiologies [9]. This is a promising finding given the fact that women with these aetiologies will often need surgery, which in many low-resource countries may not be feasible or may be delayed due to personnel or logistic barriers. The higher mortality in the NASG phase may be because of the worse condition of women on study entry. However, this finding is based on a sample size too small to draw meaningful conclusions; it may be due to chance. 

There were some limitations in the study. The first limitation relates to the before-after nonrandomized design. This may allow subjective selection of eligible cases by the physicians. However, as the conditions of patients on study entry in the NASG phase were worse, this is unlikely. Some cases of obstetric haemorrhage at the facilities may not have been enrolled, as some providers did not participate in the study, but such missed cases would have been very small in number. Further, hospital level statistics for both sites showed that no mortality or severe morbidity was missed, so it is unlikely that the effectiveness of the NASG is over-estimated. 

The results regarding blood loss confirm the previous findings that the NASG reduces blood loss in obstetric haemorrhage [5]. The effect on revealed blood loss reported in this study was 113 cc difference, which may not be clinically significant. However, this may partially explain the beneficial effect of the NASG on patient outcomes. On the other hand, this positive effect on the general outlook of the patients may have a negative effect on the speed of resuscitation. The fact that women in the NASG phase received less IV fluids and blood in the first hour may be explained by complacence in response to the improvement in the general conditions as a result of the device. Physicians may have been in less haste to infuse fluids and give blood after seeing the beneficial effect of the NASG on the vital signs, level of consciousness, and decreased blood loss. This has serious implications for training on the introduction of the NASG. Providing inadequate resuscitation or delaying management may adversely affect outcomes for women in the NASG. 

The lack of statistical significance of the difference between EAO during the two phases might be due to a lack of power of the subanalysis of nonatonic aetiologies. The sample size calculation was done to power the full study, which included all obstetric haemorrhage aetiologies, to detect differences in the outcomes. The power calculation conducted with Epi Info Version 6 showed that the study would need to include 2584 women in each arm to have 80% power and 95% confidence to detect a 1.5% difference in EAOs. Another possibility would be the worse condition on admission in the NASG group. A third explanation may be the less timely use of resuscitation received by women in the NASG phase. This question remains largely unanswered and requires a study that is powered for these aetiologies and in which treatment would begin rapidly in both phases.

## 5. Conclusion

The NASG may be a promising first aid device in the management of obstetric haemorrhage due to nonatonic aetiologies in tertiary level facilities in low-resource settings. The promise of the NASG can only be realized along with rapid implementation of shock and haemorrhage protocols. In the absence of other advances to manage these conditions, it is worthwhile to study the effectiveness of the NASG for nonatonic aetiologies in a study powered to demonstrate significant differences in outcomes.

## Figures and Tables

**Table 1:** Demographics, diagnoses, and condition on entry to study, by phase (*N* = 434).

	Pre (*N* = 226)	Post (*N* = 208)	Statistical test
Demographics			
Mean age (SD) (*n* = 433)	29.2	29.0	*t*-test, *t* = 0.47
	SD = 6.5	SD = 6.3	*P* = 0.639
Mean parity (SD) (*n* = 433)	2.4	2.5	*t*-test, *t* = −0.42
	SD = 2.2	SD = 2.2	*P* = 0.678
Mean duration of pregnancy (SD) (*n* = 388)*	24.4	25.5	*t*-test, *t* = −0.76
	SD = 14.0	SD = 14.5	*P* = 0.447

Primary definitive diagnosis, *n* (%)^‡^			

Ectopic pregnancy (*n*)	35.4% (80)	34.1% (71)	*χ* ^2^ = 0.076
			*P* = 0.782
Molar pregnancy (*n*)	3.1% (7)	4.3% (9)	*χ* ^2^ = 0.461
			*P* = 0.497
Placenta praevia (*n*)	10.2% (23)	3.9% (8)	*χ* ^2^ = 6.545
			*P* = 0.014*^*√*^*
Placenta accreta (*n*)	2.2% (5)	3.4% (7)	*χ* ^2^ = 0.536
			*P* = 0.563^*√*^
Abruption of placenta (*n*)	26.1% (59)	25.0% (52)	*χ* ^2^ = 0.070
			*P* = 0.792
Ruptured uterus (*n*)	13.3% (30)	8.7% (18)	*χ* ^2^ = 2.351
			*P* = 0.125
Vaginal/cervical lacerations (*n*)	9.7% (22)	20.7% (43)	*χ* ^2^ = 10.178
			*P* = 0.001

Condition on study entry			

Mean-estimated revealed blood loss in mL ^|| ^ (SD) (*n* = 218)^†^	1006	1219	*t*-test, *t* = −3.061
	SD = 512	SD = 514	*P* = 0.003
Mean-estimated concealed blood loss in mL (SD) (*n* = 290)^†^	1198	1324	*t*-test, *t* = −2.765
	SD = 391.1	SD = 378.4	*P* = 0.006
Women with MAP < 60 % (*n*) (*n* = 432)**	15.6% (35)	24.5% (51)	*χ* ^2^ = 5.546
			*P* = 0.020

*Does not include preterm pregnancies. ^‡^Woman could have multiple diagnoses, percentages add to more than 100%. ^*√*^Fisher's exact values reported when variables have any cell less than 10. ^ ||^Only for women with external blood loss at study admission. ^†^Women can have both revealed and concealed blood loss. **Includes women with nonpalpable blood pressure.

**Table 2:** Treatment, by phase (*N* = 434).

	Pre (*N* = 226)	Post (*N* = 208)	Statistical test
Women who received ≥1500 mL of IV fluids in the first hour from study admission % (*n*)^Φ^	93.4% (211)	64.9% (135)	*χ* ^2^ = 54.268
			*P* < 0.0001
Women who received a blood transfusion some time after study admission % (*n*)	100% (226)	99.5% (207)	*χ* ^2^ = 1.089
			*P* = 0.479^*√*^
Women who received a blood transfusion in the 1st hour from study admission % (*n*)	96.5% (218)	86.5% (180)	*χ* ^2^ = 14.017
			*P* < 0.0001^*√*^

^Φ^The protocol asked for 1500 mL to be administered in the first hour of resuscitation, however, in some cases only 1000 mL were administered in the first hour, while the remaining 500 mL were administered in the second hour. ^*√*^Fisher's exact test was used.

**Table 3:** Outcomes, by phase (*N* = 434).

	Pre (*N* = 226)	Post (*N* = 208)	Statistical test
Mean blood loss as measured in the drape (mL), (SD) (*n* = 155)^¶^	370.4	257.7	*t*-test, *t* = 4.394
	SD = 174.1	SD = 140.8	*P* < 0.0001
Mortality, % (*n*) (*n* = 434)	0.4% (1)	1.9% (4)	RR = 4.35
			95% CI = 0.49–38.57
Severe morbidity, % (*n*) (*n* = 429)*	4.0% (9)	1.0% (2)	RR = 0.25
			95% CI = 0.05–1.12
Extreme adverse outcome (EAO)**, % (*n*) (*n* = 434)	4.4% (10)	2.9% (6)	RR = 0.65
			95% CI = 0.24 – 1.76

^¶^For cases in whom the blood collection drape was used and there were data for blood loss. Women with a primary diagnosis of abruption were excluded from this analysis due to the nature of the blood loss of this aetiology-pooled internal blood loss and clots captured in the drape after delivery of the placenta which should not be included in the blood loss calculation. ^§^Only for women with definitive diagnoses of abruption of placenta, ruptured uterus, placenta previa, and placenta accrete. *Only for women who survived **EAO = mortality or morbidity.

**Table 4:** Potential side effects of NASG treatment, by phase (*N* = 433).

	Pre (*N* = 225)	Post (*N* = 208)	RR and 95% CI
Any side effects, % (*n*)	64.4% (145)	67.8% (141)	RR = 1.05
			95% CI = 0.92–1.20
Respiratory symptoms/dyspnoea, % (*n*)	7.1% (16)	8.2% (17)	RR = 1.15
			95% CI = 0.60–2.22
Reduced urine output, % (*n*)	9.8% (22)	10.1% (21)	RR = 1.03
			95% CI = 0.59–1.82
Nausea, % (*n*)	28.0% (63)	19.2% (40)	RR = 0.69
			95% CI = 0.48–0.97
Vomiting, % (*n*)	29.3% (66)	19.7% (41)	RR = 0.67
			95% CI = 0.48–0.95
Abdominal pain, % (*n*)	60.9% (137)	60.6% (126)	RR = 0.99
			95% CI = 0.85–1.16
